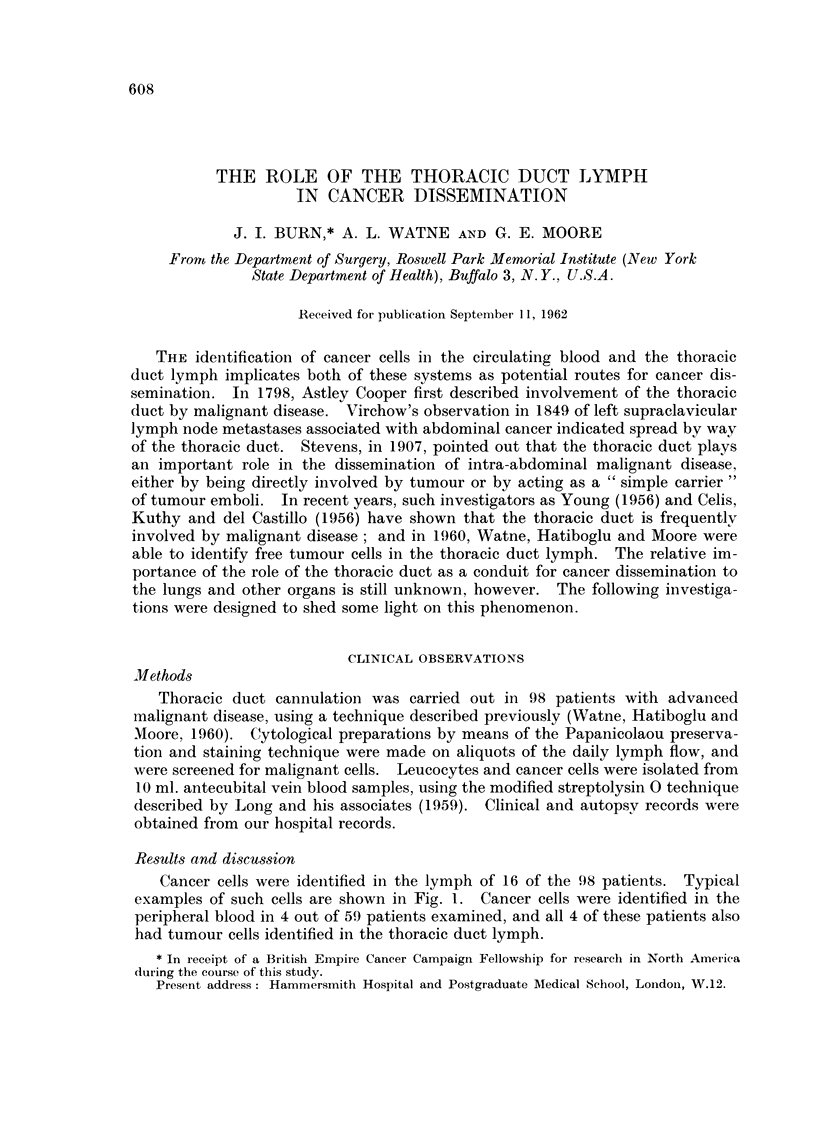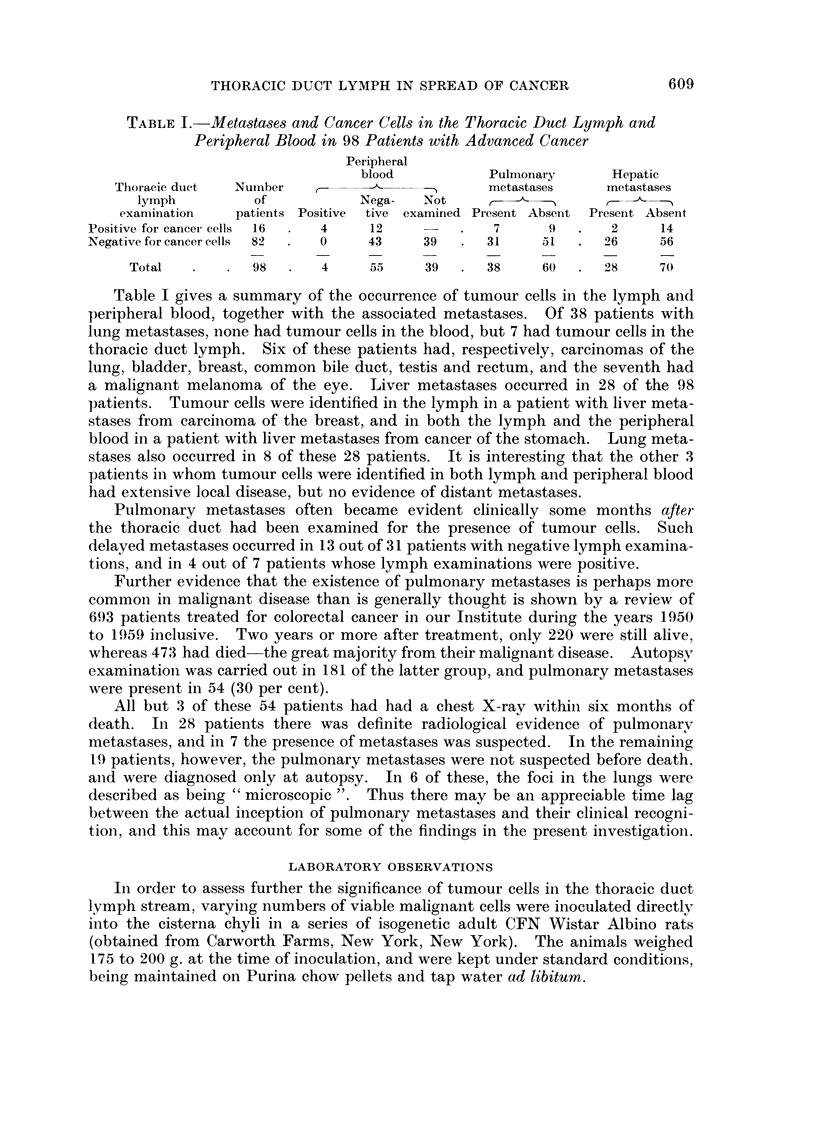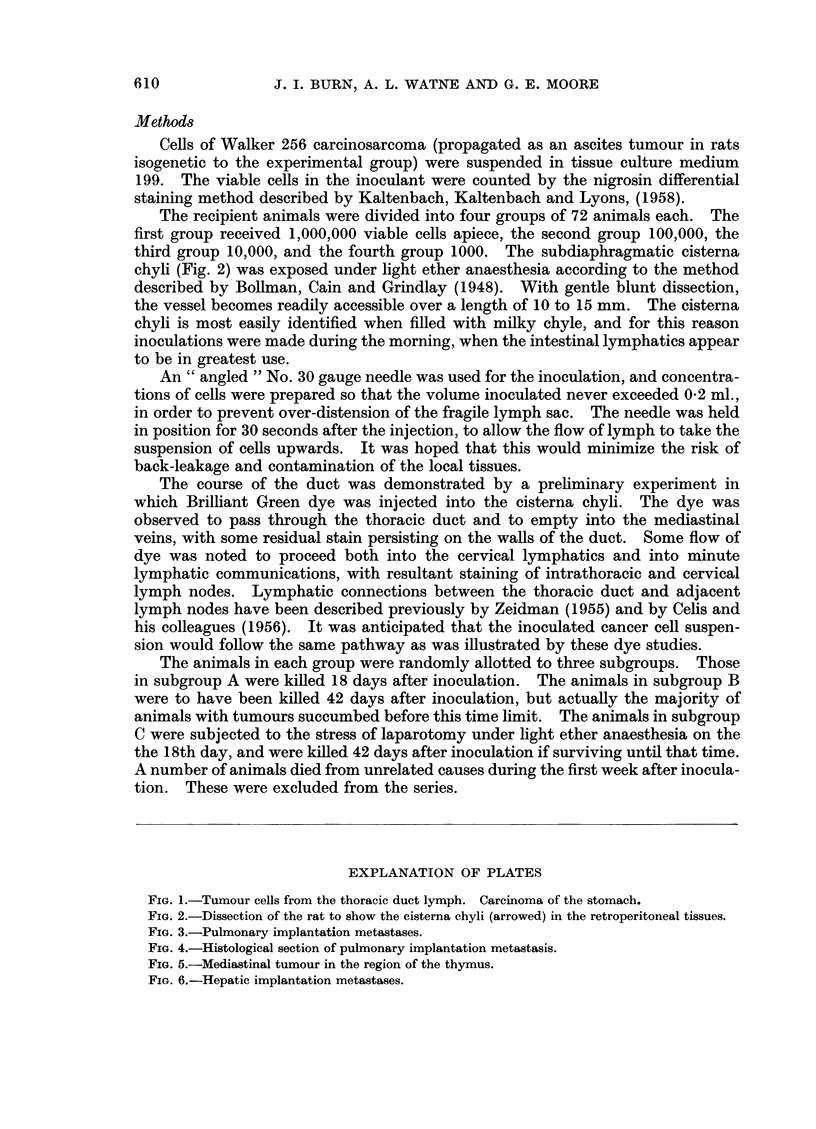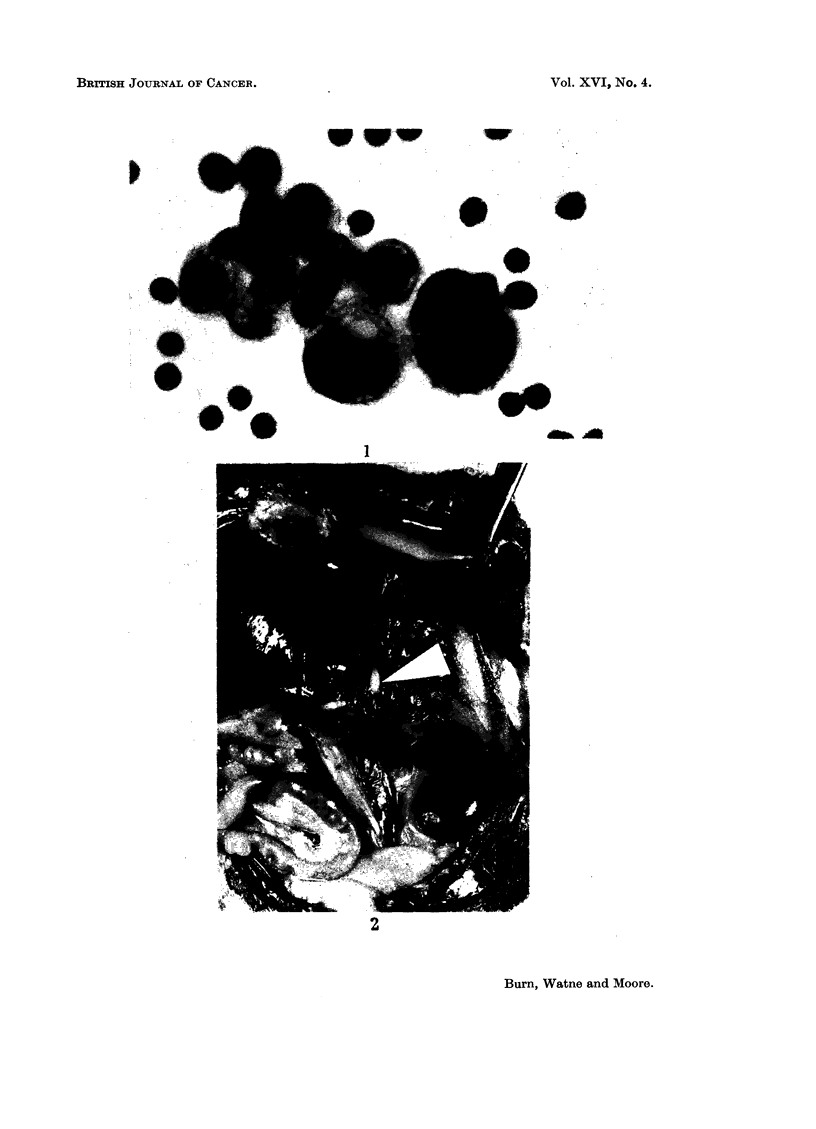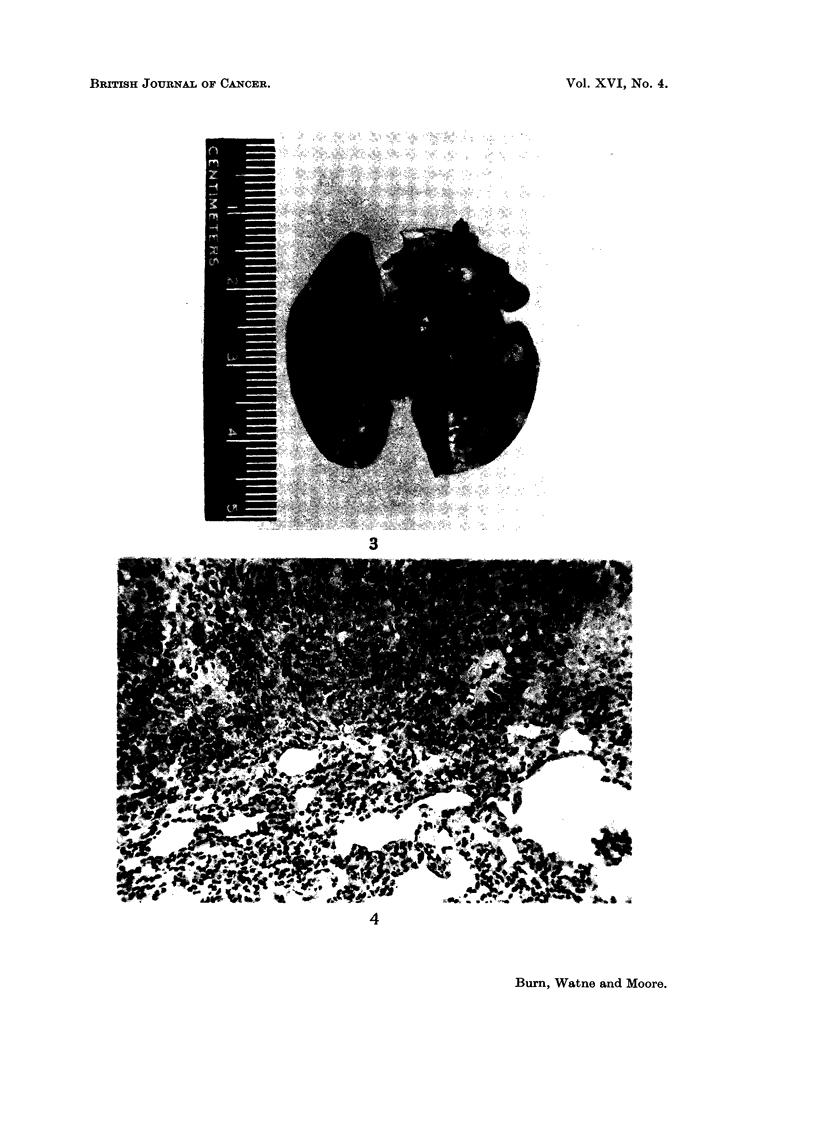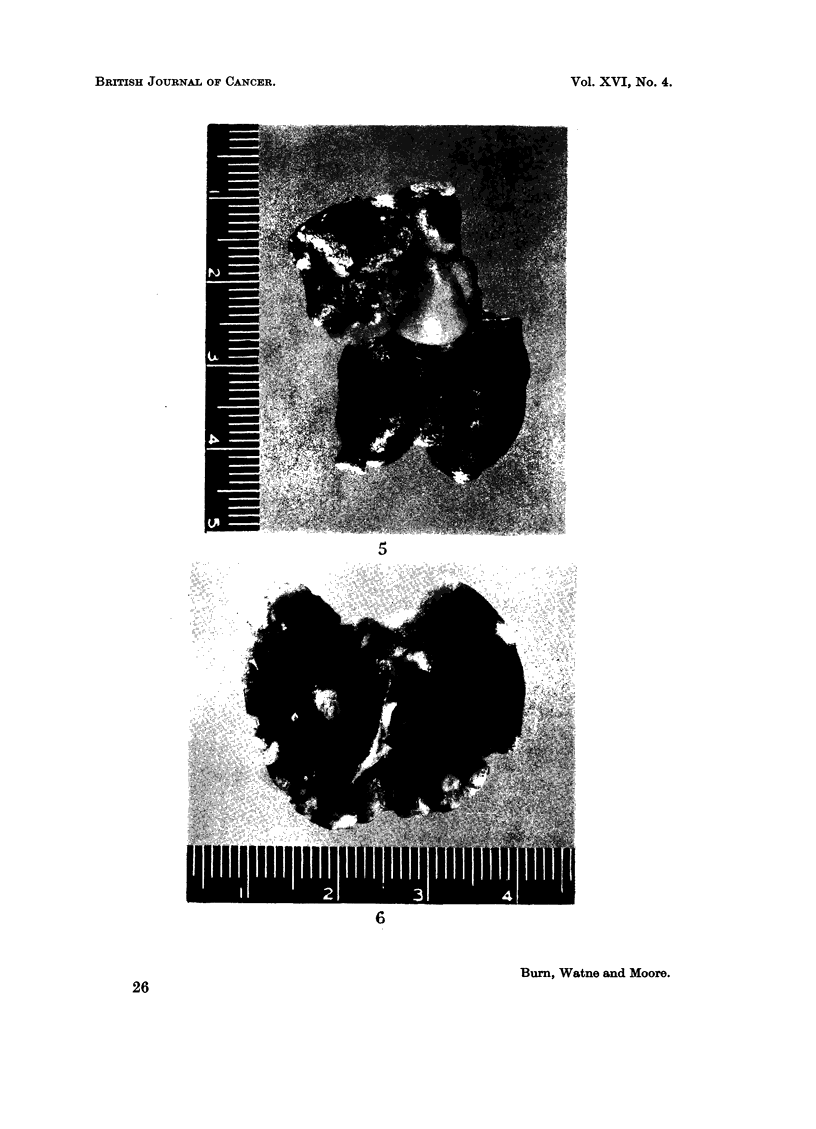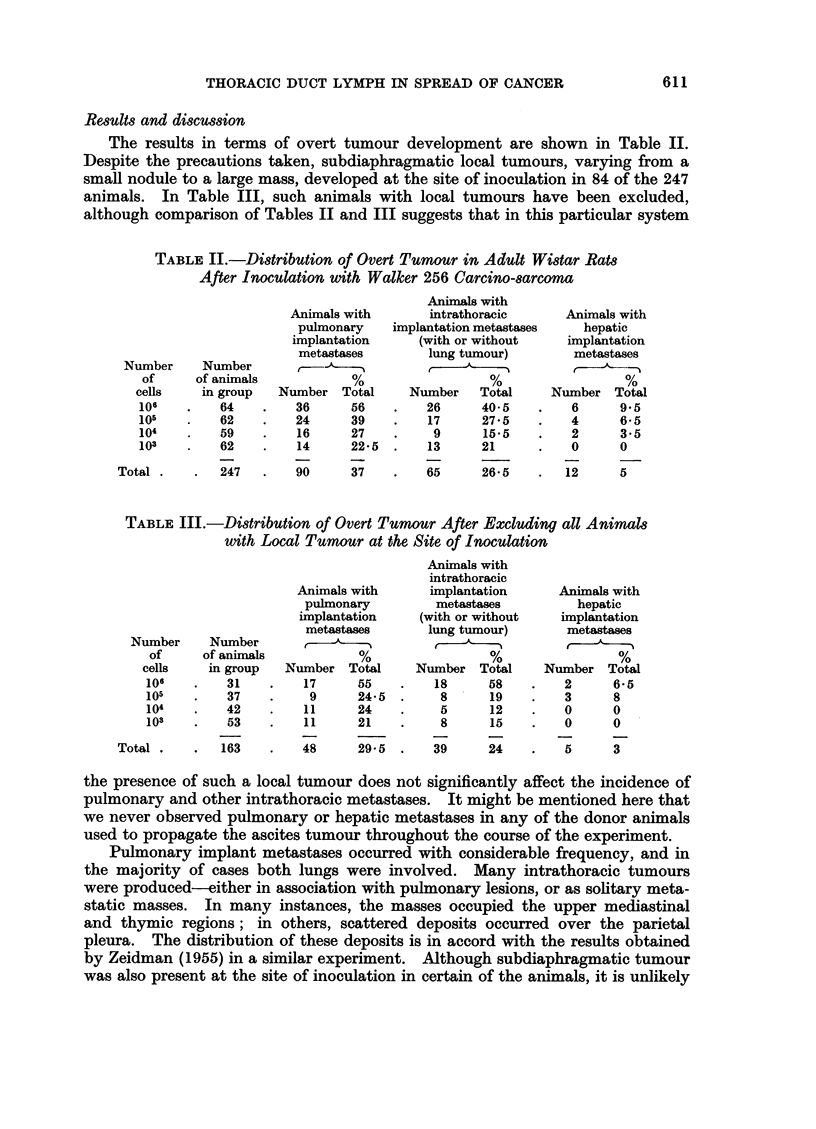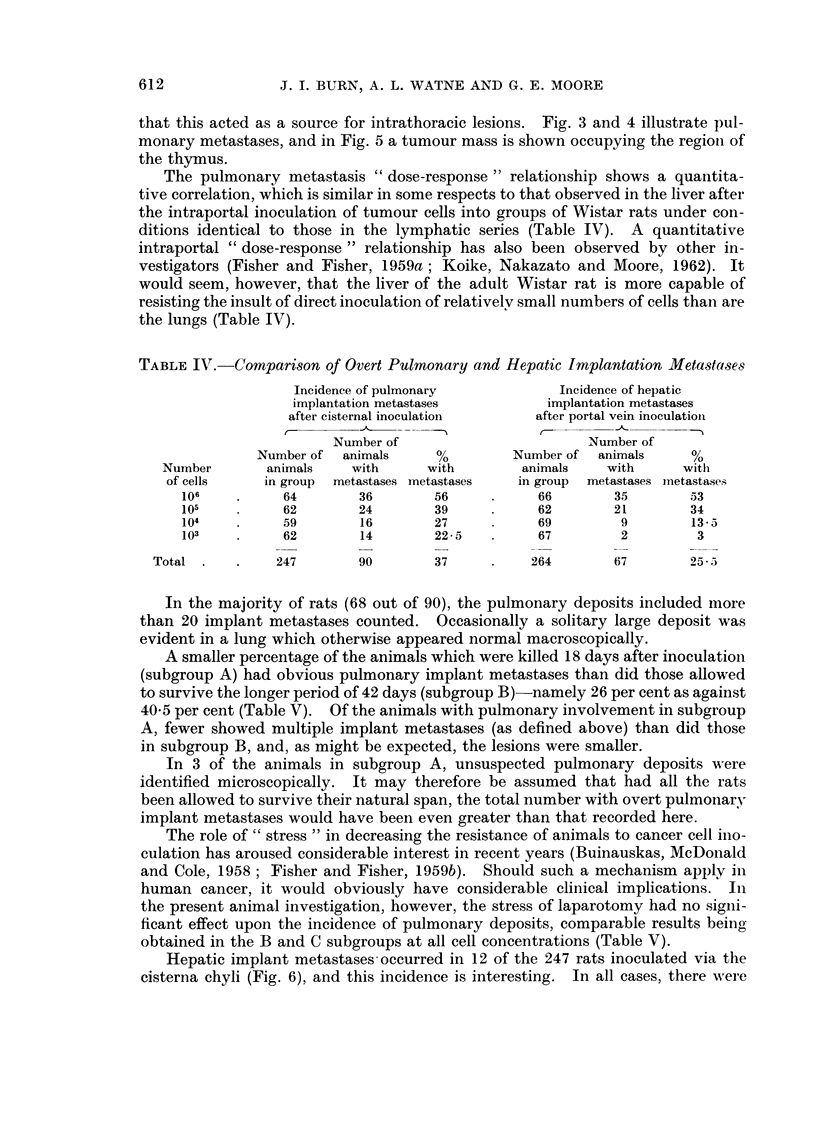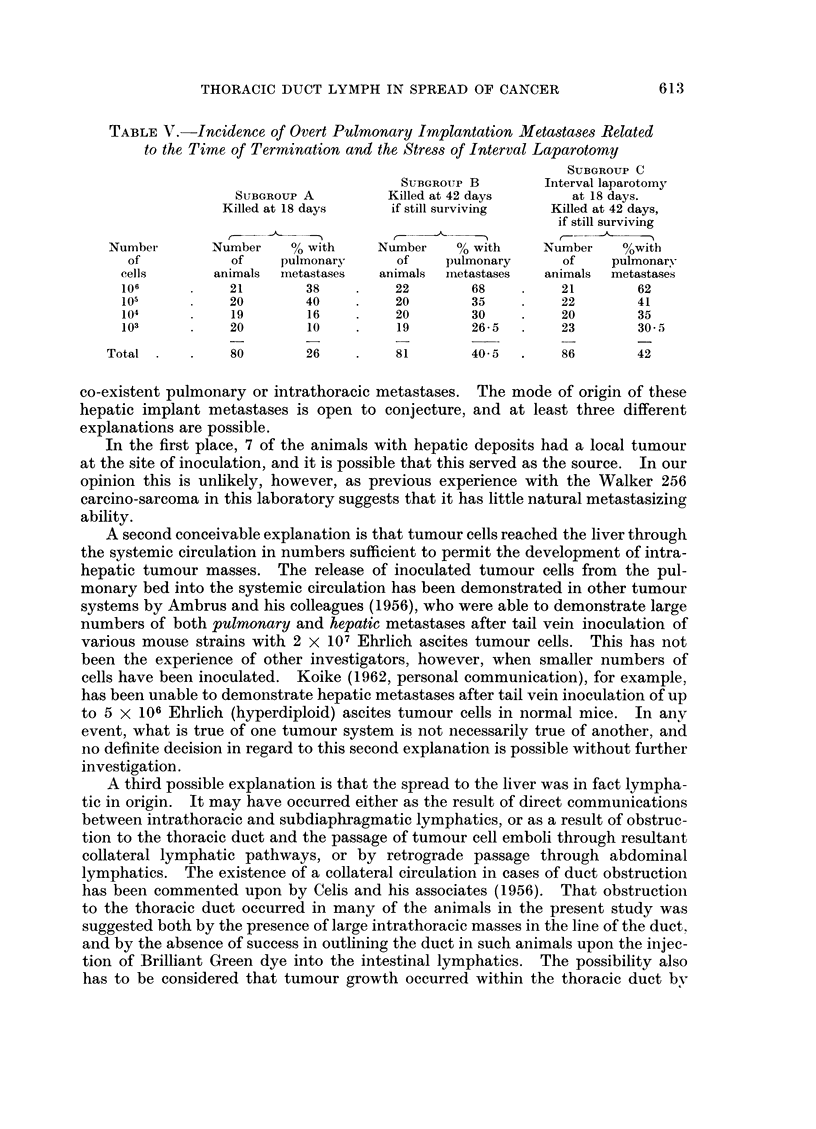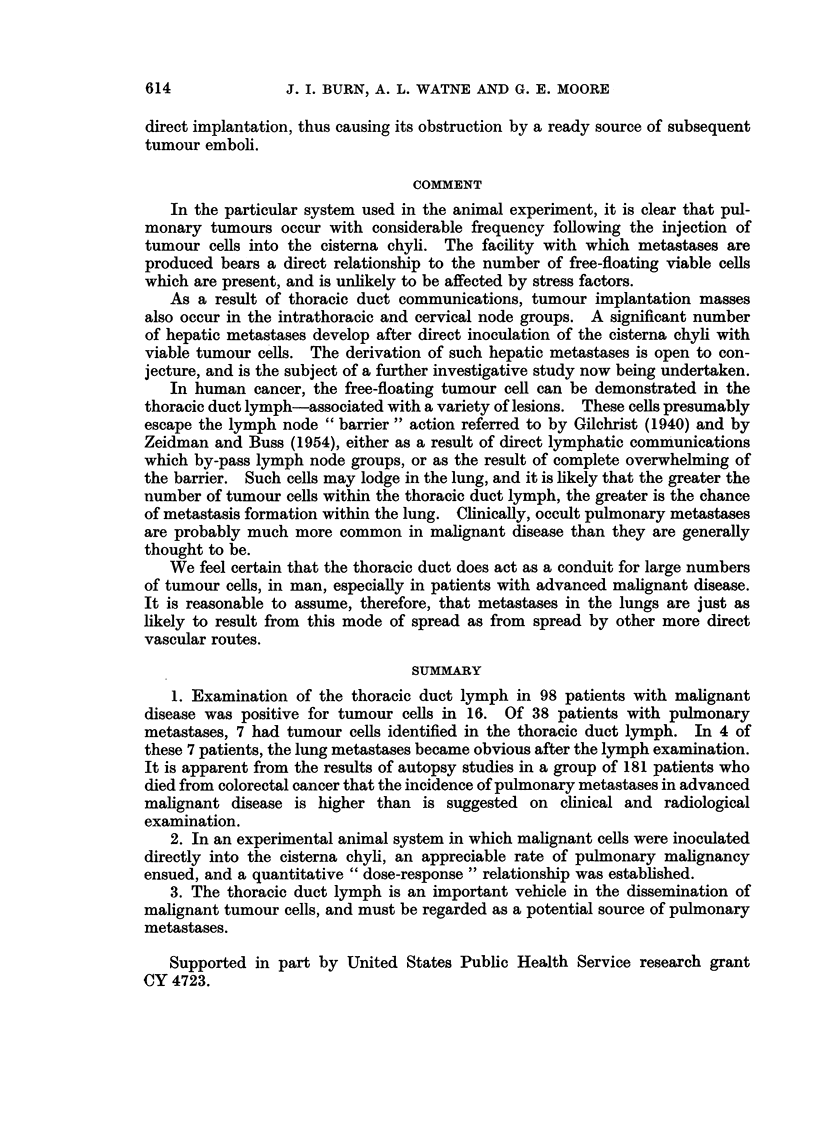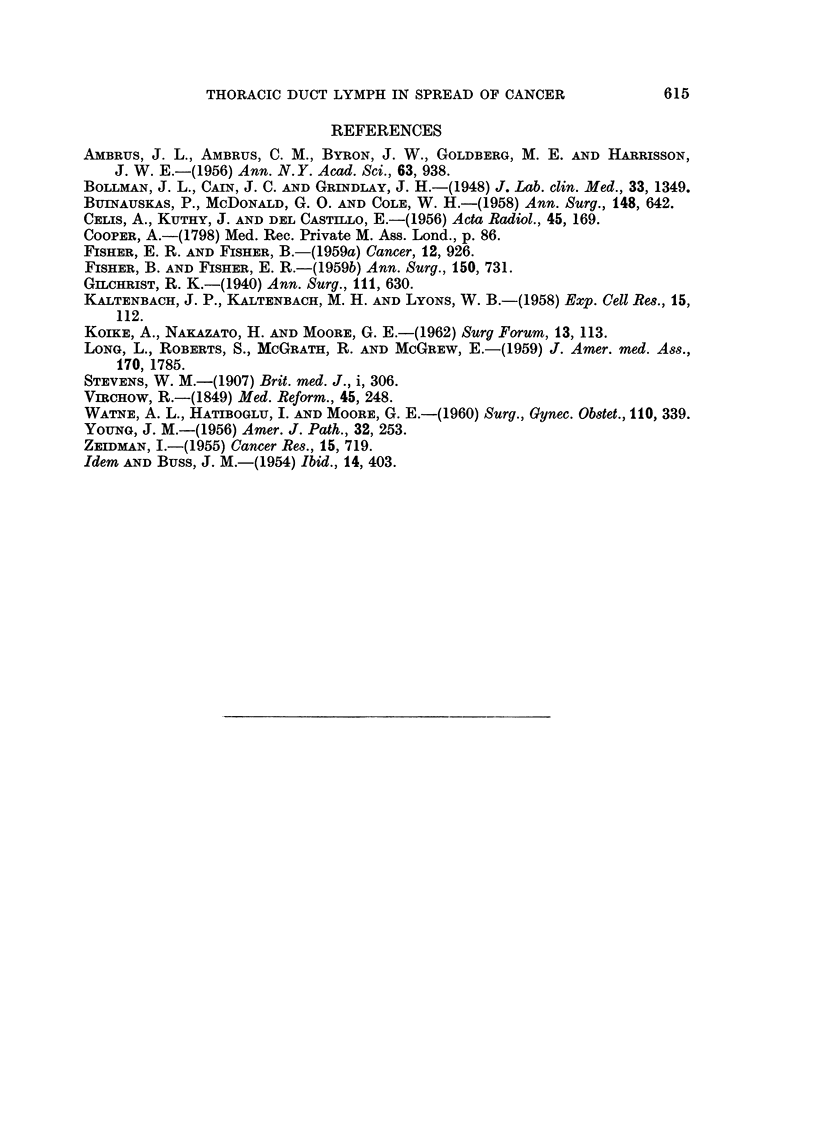# The Role of the Thoracic Duct Lymph in Cancer Dissemination

**DOI:** 10.1038/bjc.1962.71

**Published:** 1962-12

**Authors:** J. I. Burn, A. L. Watne, G. E. Moore

## Abstract

**Images:**


### THE ROLE OF THE THORACIC DUCT LYMPH IN CANCER DISSEMINATION

J. I. BURN,* A. L. WATNE AND G. E. MOORE

*From the Department of Surgery, Roswell Park Memorial Institute (New York State Department of Health), Buffalo 3, N.Y., U.S.A.*

Received for publication September 11, 1962

THE identification of cancer cells in the circulating blood and the thoracic duct lymph implicates both of these systems as potential routes for cancer dissemination. In 1798, Astley Cooper first described involvement of the thoracic duct by malignant disease. Virchow's observation in 1849 of left supraclavicular lymph node metastases associated with abdominal cancer indicated spread by way of the thoracic duct. Stevens, in 1907, pointed out that the thoracic duct plays an important role in the dissemination of intra-abdominal malignant disease, either by being directly involved by tumour or by acting as a " simple carrier " of tumour emboli. In recent years, such investigators as Young (1956) and Celis, Kuthy and del Castillo (1956) have shown that the thoracic duct is frequently involved by malignant disease ; and in 1960, Watne, Hatiboglu and Moore were able to identify free tumour cells in the thoracic duct lymph. The relative importance of the role of the thoracic duct as a conduit for cancer dissemination to the lungs and other organs is still unknown, however. The following investigations were designed to shed some light on this phenomenon.

### CLINICAL OBSERVATIONS

### *Methods*

Thoracic duct cannulation was carried out in 98 patients with advanced malignant disease, using a technique described previously (Watne, Hatiboglu and Moore, 1960). Cytological preparations by means of the Papanicolaou preservation and staining technique were made on aliquots of the daily lymph flow, and were screened for malignant cells. Leucocytes and cancer cells were isolated from 10 ml. antecubital vein blood samples, using the modified streptolysin O technique described by Long and his associates (1959). Clinical and autopsy records were obtained from our hospital records.

### *Results and discussion*

Cancer cells were identified in the lymph of 16 of the 98 patients. Typical examples of such cells are shown in Fig. 1. Cancer cells were identified in the peripheral blood in 4 out of 59 patients examined, and all 4 of these patients also had tumour cells identified in the thoracic duct lymph.

* In receipt of a British Empire Cancer Campaign Fellowship for research in North America during the course of this study.

Present address : Hammersmith Hospital and Postgraduate Medical School, London, W.12.

TABLE I.—*Metastases and Cancer Cells in the Thoracic Duct Lymph and Peripheral Blood in 98 Patients with Advanced Cancer*

| Thoracic duct lymph examination | Number of patients | Peripheral blood: Positive | Peripheral blood: Negative | Peripheral blood: Not examined | Pulmonary metastases: Present | Pulmonary metastases: Absent | Hepatic metastases: Present | Hepatic metastases: Absent |
|---|---|---|---|---|---|---|---|---|
| Positive for cancer cells | 16 | 4 | 12 | — | 7 | 9 | 2 | 14 |
| Negative for cancer cells | 82 | 0 | 43 | 39 | 31 | 51 | 26 | 56 |
| Total | 98 | 4 | 55 | 39 | 38 | 60 | 28 | 70 |

Table I gives a summary of the occurrence of tumour cells in the lymph and peripheral blood, together with the associated metastases. Of 38 patients with lung metastases, none had tumour cells in the blood, but 7 had tumour cells in the thoracic duct lymph. Six of these patients had, respectively, carcinomas of the lung, bladder, breast, common bile duct, testis and rectum, and the seventh had a malignant melanoma of the eye. Liver metastases occurred in 28 of the 98 patients. Tumour cells were identified in the lymph in a patient with liver metastases from carcinoma of the breast, and in both the lymph and the peripheral blood in a patient with liver metastases from cancer of the stomach. Lung metastases also occurred in 8 of these 28 patients. It is interesting that the other 3 patients in whom tumour cells were identified in both lymph and peripheral blood had extensive local disease, but no evidence of distant metastases.

Pulmonary metastases often became evident clinically some months *after* the thoracic duct had been examined for the presence of tumour cells. Such delayed metastases occurred in 13 out of 31 patients with negative lymph examinations, and in 4 out of 7 patients whose lymph examinations were positive.

Further evidence that the existence of pulmonary metastases is perhaps more common in malignant disease than is generally thought is shown by a review of 693 patients treated for colorectal cancer in our Institute during the years 1950 to 1959 inclusive. Two years or more after treatment, only 220 were still alive, whereas 473 had died—the great majority from their malignant disease. Autopsy examination was carried out in 181 of the latter group, and pulmonary metastases were present in 54 (30 per cent).

All but 3 of these 54 patients had had a chest X-ray within six months of death. In 28 patients there was definite radiological evidence of pulmonary metastases, and in 7 the presence of metastases was suspected. In the remaining 19 patients, however, the pulmonary metastases were not suspected before death, and were diagnosed only at autopsy. In 6 of these, the foci in the lungs were described as being " microscopic ". Thus there may be an appreciable time lag between the actual inception of pulmonary metastases and their clinical recognition, and this may account for some of the findings in the present investigation.

### LABORATORY OBSERVATIONS

In order to assess further the significance of tumour cells in the thoracic duct lymph stream, varying numbers of viable malignant cells were inoculated directly into the cisterna chyli in a series of isogenetic adult CFN Wistar Albino rats (obtained from Carworth Farms, New York, New York). The animals weighed 175 to 200 g. at the time of inoculation, and were kept under standard conditions, being maintained on Purina chow pellets and tap water *ad libitum*.

*Methods*

Cells of Walker 256 carcinosarcoma (propagated as an ascites tumour in rats isogenetic to the experimental group) were suspended in tissue culture medium 199. The viable cells in the inoculant were counted by the nigrosin differential staining method described by Kaltenbach, Kaltenbach and Lyons, (1958).

The recipient animals were divided into four groups of 72 animals each. The first group received 1,000,000 viable cells apiece, the second group 100,000, the third group 10,000, and the fourth group 1000. The subdiaphragmatic cisterna chyli (Fig. 2) was exposed under light ether anaesthesia according to the method described by Bollman, Cain and Grindlay (1948). With gentle blunt dissection, the vessel becomes readily accessible over a length of 10 to 15 mm. The cisterna chyli is most easily identified when filled with milky chyle, and for this reason inoculations were made during the morning, when the intestinal lymphatics appear to be in greatest use.

An " angled " No. 30 gauge needle was used for the inoculation, and concentrations of cells were prepared so that the volume inoculated never exceeded 0·2 ml., in order to prevent over-distension of the fragile lymph sac. The needle was held in position for 30 seconds after the injection, to allow the flow of lymph to take the suspension of cells upwards. It was hoped that this would minimize the risk of back-leakage and contamination of the local tissues.

The course of the duct was demonstrated by a preliminary experiment in which Brilliant Green dye was injected into the cisterna chyli. The dye was observed to pass through the thoracic duct and to empty into the mediastinal veins, with some residual stain persisting on the walls of the duct. Some flow of dye was noted to proceed both into the cervical lymphatics and into minute lymphatic communications, with resultant staining of intrathoracic and cervical lymph nodes. Lymphatic connections between the thoracic duct and adjacent lymph nodes have been described previously by Zeidman (1955) and by Celis and his colleagues (1956). It was anticipated that the inoculated cancer cell suspension would follow the same pathway as was illustrated by these dye studies.

The animals in each group were randomly allotted to three subgroups. Those in subgroup A were killed 18 days after inoculation. The animals in subgroup B were to have been killed 42 days after inoculation, but actually the majority of animals with tumours succumbed before this time limit. The animals in subgroup C were subjected to the stress of laparotomy under light ether anaesthesia on the the 18th day, and were killed 42 days after inoculation if surviving until that time. A number of animals died from unrelated causes during the first week after inoculation. These were excluded from the series.

---

EXPLANATION OF PLATES

FIG. 1.—Tumour cells from the thoracic duct lymph. Carcinoma of the stomach.

FIG. 2.—Dissection of the rat to show the cisterna chyli (arrowed) in the retroperitoneal tissues.

FIG. 3.—Pulmonary implantation metastases.

FIG. 4.—Histological section of pulmonary implantation metastasis.

FIG. 5.—Mediastinal tumour in the region of the thymus.

FIG. 6.—Hepatic implantation metastases.

1

2

Burn, Watne and Moore.

3

4

5

6

Burn, Watne and Moore.

*Results and discussion*

The results in terms of overt tumour development are shown in Table II. Despite the precautions taken, subdiaphragmatic local tumours, varying from a small nodule to a large mass, developed at the site of inoculation in 84 of the 247 animals. In Table III, such animals with local tumours have been excluded, although comparison of Tables II and III suggests that in this particular system

TABLE II.—*Distribution of Overt Tumour in Adult Wistar Rats After Inoculation with Walker* 256 *Carcino-sarcoma*

| Number of cells | Number of animals in group | Animals with pulmonary implantation metastases: Number | % Total | Animals with intrathoracic implantation metastases (with or without lung tumour): Number | % Total | Animals with hepatic implantation metastases: Number | % Total |
|---|---|---|---|---|---|---|---|
| $10^6$ | 64 | 36 | 56 | 26 | 40·5 | 6 | 9·5 |
| $10^5$ | 62 | 24 | 39 | 17 | 27·5 | 4 | 6·5 |
| $10^4$ | 59 | 16 | 27 | 9 | 15·5 | 2 | 3·5 |
| $10^3$ | 62 | 14 | 22·5 | 13 | 21 | 0 | 0 |
| Total | 247 | 90 | 37 | 65 | 26·5 | 12 | 5 |

TABLE III.—*Distribution of Overt Tumour After Excluding all Animals with Local Tumour at the Site of Inoculation*

| Number of cells | Number of animals in group | Animals with pulmonary implantation metastases: Number | % Total | Animals with intrathoracic implantation metastases (with or without lung tumour): Number | % Total | Animals with hepatic implantation metastases: Number | % Total |
|---|---|---|---|---|---|---|---|
| $10^6$ | 31 | 17 | 55 | 18 | 58 | 2 | 6·5 |
| $10^5$ | 37 | 9 | 24·5 | 8 | 19 | 3 | 8 |
| $10^4$ | 42 | 11 | 24 | 5 | 12 | 0 | 0 |
| $10^3$ | 53 | 11 | 21 | 8 | 15 | 0 | 0 |
| Total | 163 | 48 | 29·5 | 39 | 24 | 5 | 3 |

the presence of such a local tumour does not significantly affect the incidence of pulmonary and other intrathoracic metastases. It might be mentioned here that we never observed pulmonary or hepatic metastases in any of the donor animals used to propagate the ascites tumour throughout the course of the experiment.

Pulmonary implant metastases occurred with considerable frequency, and in the majority of cases both lungs were involved. Many intrathoracic tumours were produced—either in association with pulmonary lesions, or as solitary metastatic masses. In many instances, the masses occupied the upper mediastinal and thymic regions ; in others, scattered deposits occurred over the parietal pleura. The distribution of these deposits is in accord with the results obtained by Zeidman (1955) in a similar experiment. Although subdiaphragmatic tumour was also present at the site of inoculation in certain of the animals, it is unlikely that this acted as a source for intrathoracic lesions. Fig. 3 and 4 illustrate pulmonary metastases, and in Fig. 5 a tumour mass is shown occupying the region of the thymus.

The pulmonary metastasis " dose-response " relationship shows a quantitative correlation, which is similar in some respects to that observed in the liver after the intraportal inoculation of tumour cells into groups of Wistar rats under conditions identical to those in the lymphatic series (Table IV). A quantitative intraportal " dose-response " relationship has also been observed by other investigators (Fisher and Fisher, 1959*a* ; Koike, Nakazato and Moore, 1962). It would seem, however, that the liver of the adult Wistar rat is more capable of resisting the insult of direct inoculation of relatively small numbers of cells than are the lungs (Table IV).

TABLE IV.—*Comparison of Overt Pulmonary and Hepatic Implantation Metastases*

| Number of cells | Incidence of pulmonary implantation metastases after cisternal inoculation: Number of animals in group | Number of animals with metastases | % with metastases | Incidence of hepatic implantation metastases after portal vein inoculation: Number of animals in group | Number of animals with metastases | % with metastases |
|---|---|---|---|---|---|---|
| $10^6$ | 64 | 36 | 56 | 66 | 35 | 53 |
| $10^5$ | 62 | 24 | 39 | 62 | 21 | 34 |
| $10^4$ | 59 | 16 | 27 | 69 | 9 | 13·5 |
| $10^3$ | 62 | 14 | 22·5 | 67 | 2 | 3 |
| Total | 247 | 90 | 37 | 264 | 67 | 25·5 |

In the majority of rats (68 out of 90), the pulmonary deposits included more than 20 implant metastases counted. Occasionally a solitary large deposit was evident in a lung which otherwise appeared normal macroscopically.

A smaller percentage of the animals which were killed 18 days after inoculation (subgroup A) had obvious pulmonary implant metastases than did those allowed to survive the longer period of 42 days (subgroup B)—namely 26 per cent as against 40·5 per cent (Table V). Of the animals with pulmonary involvement in subgroup A, fewer showed multiple implant metastases (as defined above) than did those in subgroup B, and, as might be expected, the lesions were smaller.

In 3 of the animals in subgroup A, unsuspected pulmonary deposits were identified microscopically. It may therefore be assumed that had all the rats been allowed to survive their natural span, the total number with overt pulmonary implant metastases would have been even greater than that recorded here.

The role of " stress " in decreasing the resistance of animals to cancer cell inoculation has aroused considerable interest in recent years (Buinauskas, McDonald and Cole, 1958 ; Fisher and Fisher, 1959*b*). Should such a mechanism apply in human cancer, it would obviously have considerable clinical implications. In the present animal investigation, however, the stress of laparotomy had no significant effect upon the incidence of pulmonary deposits, comparable results being obtained in the B and C subgroups at all cell concentrations (Table V).

Hepatic implant metastases occurred in 12 of the 247 rats inoculated via the cisterna chyli (Fig. 6), and this incidence is interesting. In all cases, there were

TABLE V.—*Incidence of Overt Pulmonary Implantation Metastases Related to the Time of Termination and the Stress of Interval Laparotomy*

| | SUBGROUP A Killed at 18 days | | SUBGROUP B Killed at 42 days if still surviving | | SUBGROUP C Interval laparotomy at 18 days. Killed at 42 days, if still surviving | |
|---|---|---|---|---|---|---|
| Number of cells | Number of animals | % with pulmonary metastases | Number of animals | % with pulmonary metastases | Number of animals | %with pulmonary metastases |
| $10^6$ | 21 | 38 | 22 | 68 | 21 | 62 |
| $10^5$ | 20 | 40 | 20 | 35 | 22 | 41 |
| $10^4$ | 19 | 16 | 20 | 30 | 20 | 35 |
| $10^3$ | 20 | 10 | 19 | 26·5 | 23 | 30·5 |
| Total | 80 | 26 | 81 | 40·5 | 86 | 42 |

co-existent pulmonary or intrathoracic metastases. The mode of origin of these hepatic implant metastases is open to conjecture, and at least three different explanations are possible.

In the first place, 7 of the animals with hepatic deposits had a local tumour at the site of inoculation, and it is possible that this served as the source. In our opinion this is unlikely, however, as previous experience with the Walker 256 carcino-sarcoma in this laboratory suggests that it has little natural metastasizing ability.

A second conceivable explanation is that tumour cells reached the liver through the systemic circulation in numbers sufficient to permit the development of intra-hepatic tumour masses. The release of inoculated tumour cells from the pulmonary bed into the systemic circulation has been demonstrated in other tumour systems by Ambrus and his colleagues (1956), who were able to demonstrate large numbers of both *pulmonary* and *hepatic* metastases after tail vein inoculation of various mouse strains with $2 \times 10^7$ Ehrlich ascites tumour cells. This has not been the experience of other investigators, however, when smaller numbers of cells have been inoculated. Koike (1962, personal communication), for example, has been unable to demonstrate hepatic metastases after tail vein inoculation of up to $5 \times 10^6$ Ehrlich (hyperdiploid) ascites tumour cells in normal mice. In any event, what is true of one tumour system is not necessarily true of another, and no definite decision in regard to this second explanation is possible without further investigation.

A third possible explanation is that the spread to the liver was in fact lymphatic in origin. It may have occurred either as the result of direct communications between intrathoracic and subdiaphragmatic lymphatics, or as a result of obstruction to the thoracic duct and the passage of tumour cell emboli through resultant collateral lymphatic pathways, or by retrograde passage through abdominal lymphatics. The existence of a collateral circulation in cases of duct obstruction has been commented upon by Celis and his associates (1956). That obstruction to the thoracic duct occurred in many of the animals in the present study was suggested both by the presence of large intrathoracic masses in the line of the duct, and by the absence of success in outlining the duct in such animals upon the injection of Brilliant Green dye into the intestinal lymphatics. The possibility also has to be considered that tumour growth occurred within the thoracic duct by direct implantation, thus causing its obstruction by a ready source of subsequent tumour emboli.

### COMMENT

In the particular system used in the animal experiment, it is clear that pulmonary tumours occur with considerable frequency following the injection of tumour cells into the cisterna chyli. The facility with which metastases are produced bears a direct relationship to the number of free-floating viable cells which are present, and is unlikely to be affected by stress factors.

As a result of thoracic duct communications, tumour implantation masses also occur in the intrathoracic and cervical node groups. A significant number of hepatic metastases develop after direct inoculation of the cisterna chyli with viable tumour cells. The derivation of such hepatic metastases is open to conjecture, and is the subject of a further investigative study now being undertaken.

In human cancer, the free-floating tumour cell can be demonstrated in the thoracic duct lymph—associated with a variety of lesions. These cells presumably escape the lymph node " barrier " action referred to by Gilchrist (1940) and by Zeidman and Buss (1954), either as a result of direct lymphatic communications which by-pass lymph node groups, or as the result of complete overwhelming of the barrier. Such cells may lodge in the lung, and it is likely that the greater the number of tumour cells within the thoracic duct lymph, the greater is the chance of metastasis formation within the lung. Clinically, occult pulmonary metastases are probably much more common in malignant disease than they are generally thought to be.

We feel certain that the thoracic duct does act as a conduit for large numbers of tumour cells, in man, especially in patients with advanced malignant disease. It is reasonable to assume, therefore, that metastases in the lungs are just as likely to result from this mode of spread as from spread by other more direct vascular routes.

### SUMMARY

1. Examination of the thoracic duct lymph in 98 patients with malignant disease was positive for tumour cells in 16. Of 38 patients with pulmonary metastases, 7 had tumour cells identified in the thoracic duct lymph. In 4 of these 7 patients, the lung metastases became obvious after the lymph examination. It is apparent from the results of autopsy studies in a group of 181 patients who died from colorectal cancer that the incidence of pulmonary metastases in advanced malignant disease is higher than is suggested on clinical and radiological examination.

2. In an experimental animal system in which malignant cells were inoculated directly into the cisterna chyli, an appreciable rate of pulmonary malignancy ensued, and a quantitative " dose-response " relationship was established.

3. The thoracic duct lymph is an important vehicle in the dissemination of malignant tumour cells, and must be regarded as a potential source of pulmonary metastases.

Supported in part by United States Public Health Service research grant CY 4723.

### REFERENCES

AMBRUS, J. L., AMBRUS, C. M., BYRON, J. W., GOLDBERG, M. E. AND HARRISSON, J. W. E.—(1956) *Ann. N.Y. Acad. Sci.*, **63**, 938.
BOLLMAN, J. L., CAIN, J. C. AND GRINDLAY, J. H.—(1948) *J. Lab. clin. Med.*, **33**, 1349.
BUINAUSKAS, P., MCDONALD, G. O. AND COLE, W. H.—(1958) *Ann. Surg.*, **148**, 642.
CELIS, A., KUTHY, J. AND DEL CASTILLO, E.—(1956) *Acta Radiol.*, **45**, 169.
COOPER, A.—(1798) Med. Rec. Private M. Ass. Lond., p. 86.
FISHER, E. R. AND FISHER, B.—(1959*a*) *Cancer*, **12**, 926.
FISHER, B. AND FISHER, E. R.—(1959*b*) *Ann. Surg.*, **150**, 731.
GILCHRIST, R. K.—(1940) *Ann. Surg.*, **111**, 630.
KALTENBACH, J. P., KALTENBACH, M. H. AND LYONS, W. B.—(1958) *Exp. Cell Res.*, **15**, 112.
KOIKE, A., NAKAZATO, H. AND MOORE, G. E.—(1962) *Surg Forum*, **13**, 113.
LONG, L., ROBERTS, S., MCGRATH, R. AND MCGREW, E.—(1959) *J. Amer. med. Ass.*, **170**, 1785.
STEVENS, W. M.—(1907) *Brit. med. J.*, i, 306.
VIRCHOW, R.—(1849) *Med. Reform.*, **45**, 248.
WATNE, A. L., HATIBOGLU, I. AND MOORE, G. E.—(1960) *Surg., Gynec. Obstet.*, **110**, 339.
YOUNG, J. M.—(1956) *Amer. J. Path.*, **32**, 253.
ZEIDMAN, I.—(1955) *Cancer Res.*, **15**, 719.
*Idem* AND BUSS, J. M.—(1954) *Ibid.*, **14**, 403.